# Special Issue “Recent Molecular Research on Preeclampsia”

**DOI:** 10.3390/ijms262110572

**Published:** 2025-10-30

**Authors:** Natalia Ivanovna Agalakova

**Affiliations:** Sechenov Institute of Evolutionary Physiology and Biochemistry, Russian Academy of Sciences, 44 Thorez Avenue, 194223 Saint-Petersburg, Russia; nagalak@mail.ru

## 1. Introduction

Preeclampsia (PE) is a severe hypertensive disorder of pregnancy accompanied by systemic organ and vascular damage [1]. It not only remains one of the leading causes of maternal and perinatal mortality, but also provokes long-life consequences for both women and children, the most severe of which includes impacts on the cardiovascular and nervous systems [2,3]. Despite extensive research, PE etiopathogenesis is not completely understood. As a result, its prevention and treatment are limited, and delivery is still the only known cure. Therefore, the major focuses of current investigations are deciphering the molecular mechanisms underlying PE development, and identification of novel biomarkers which could improve early (first trimester) PE prediction, which is crucial for the monitoring of women with confirmed diagnoses, timely therapeutic intervention and, consequently, safe maternal and perinatal outcomes.

This Special Issue is a set of 12 research and review articles providing an overview into complex nature of PE and recent findings of circulating biomarkers. In addition, two articles of the Issue are dedicated to molecular mechanisms of fetal growth restriction (FGR)—another leading cause of stillbirth and neonatal mortality/morbidity due to failure of the fetus to achieve its genetically determined growth potential, primarily due to placental dysfunction [4]. Similarly to PE, FGR is a cause of long-term consequences for humans which include increased risk of cardiovascular and metabolic disorders in adulthood [5]. Therefore, understanding the mechanisms implicated in the development of FGR is crucial for developing targeted therapies and improving pregnancy outcomes.

## 2. Preeclampsia

As a part of the Issue, the review by Chiang and coworkers summarized currently existing PE models and recent advances in understanding the pathophysiological mechanisms underlying PE development, with a focus on placental changes; molecular and cellular effects of hormones, complements, and cytokines; and genetic factors (CircRNA, miRNA, and lncRNA); as well as on the role of currently applied medications (**Contribution 1**).

A few original papers present diverse molecular and cellular alterations responsible for PE. Shields and collaborators proposed Caspase-1 as a key contributor to PE, since its inhibition attenuated PE features in a rodent model of placental ischemia, increased the number of live pups, and reduced PE features (mean arterial pressure), placental IL-1β, inflammatory TH17, and cNK populations (**Contribution 2**). The authors also suggest that Caspase-1 might be a potential therapeutic target to improve maternal outcomes in PE.

Flores-Pliego and coworkers demonstrated that human umbilical vein endothelial cells (HUVECs) from patients with PE showed significantly higher oxidative stress and elevated levels of IL-8 and MMP-2 in comparison to cells isolated from vessels of women with healthy pregnancy. The molecular network leading to extensive PE-associated endothelial disfunction includes the abnormal release of inflammatory cytokine IL-8, which may act as a regulator of matrix metalloproteinase MMP-2 (**Contribution 3**).

Antonic and colleagues showed that the development of late PE is preceded by lower cholesterol levels and cholesterol absorption markers (campesterol and β-sitosterol) in the HDL (high-density lipoprotein) particles in the second trimester. This phenomenon was accompanied by decreased HDL phytosterol, apolipoprotein A-I (apoA-I), and paraoxonase 1 (PON1) content, which suggests impaired capacity for intestinal HDL synthesis and HDL-mediated cholesterol efflux (**Contribution 4**).

Using microfluidics techniques, Alexandrova-Watanabe and coworkers described altered rheological characteristics of erythrocytes of PE patients. The cells become more adhesive and form stable large aggregates, with this effect being partially due to the effect of oxidation. These processes are more pronounced in severe PE, preterm birth and low birth weight conditions (**Contribution 5**).

Important question on PE pathophysiology is whether it shares a common origin with other hypertensive disorders. Thus, the typical feature of both PE and cardiovascular diseases is dysregulation of hypotensive hormone ANP (atrial natriuretic peptide) and its activating protease corin. The review by Wu highlighted the importance of natriuretic peptide signaling in pregnancy and analyzed the current concepts implicating ANP and corin in uterine tissue remodeling and potentially in PE and gestational hypertension. The existing findings demonstrate that in the pregnant uterus, ANP enhances endometrial decidualization and promotes spiral artery remodeling and trophoblast invasion, while ANP deficiency impairs these processes, thus causing PE (**Contribution 6**).

However, using primary HUVECs, plasma, placentas, and omental arteries from healthy and PE pregnancies, Binder and collaborators demonstrated an absence of direct involvement of ANP to the regulation of endothelial cell proliferation/migration and markers of endothelial dysfunction. Although the expression of ANP receptors was significantly increased in PE vessels, it was not due to exposure to circulating preeclamptic toxins. In addition, the supplementation of endothelial cells with ANP did not promote proliferation or migration, nor did ANP affect the markers of endothelial dysfunction. (**Contribution 7**).

As a part of the important field on PE research, a few works of this Special Issue made attempts to identify novel PE-associated biomarkers which could be measured in maternal blood at the first trimester for early PE screening.

The retrospective case–control study of Camacho-Carrasco and coworkers demonstrated that multivariable models which combine maternal clinical variables, known placental factors (PlGF, sFlt-1, betaHCG, and PAPP-A), and novel markers of endothelial dysfunction and cell stress (cell-free DNA, cfDNA, total endothelial, and platelet microvesicles) possess acceptable-to-very good discriminative performance in respect to later development of different types of PE and PE-related maternal and fetal severe adverse events. Such an integrated screening strategy applied in the first trimester might improve risk stratification for PE (especially for late PE) and severe maternal or fetal complications (**Contribution 8**).

A pilot study of maternal serum proteome at 11-13 weeks of gestation performed by Starodubseva and colleagues identified 10 proteins whose early changes correlate with four subtypes of PE. These proteins are implicated in critical molecular processes, such as complement and coagulation cascades, platelet activation, and insulin-like growth factor pathways. The authors suggest that serum-based proteomic profiling possesses higher predictive efficacy for early detection of PE than traditional first-trimester screening methods (**Contribution 9**).

Using transcriptomic dataset analysis, Hirata and collaborators demonstrated that the combination of 12 differentially expressed olfactory receptor (OR) genes have a high prognostic power for later PE development before 20 weeks of gestation, while 7 ORs showed the capacity to predict early PE. The authors suggest that differentially expressed ORs might be used as biomarkers to predict PE at the beginning of pregnancy (**Contribution 10**).

The work of Liu and coworkers found positive associations between systolic blood pressure during late gestation and elevated levels of Apolipoprotein A1 (APOA1) in the plasma and placental tissues of PE patients. Although an addition of APO1 to traditional sFlt-1/PlGF screening combination did not increase the predictive efficiency of PE in either early- or mid-term gestation, the authors proposed APOA1 as a marker of the severity of the disease (**Contribution 11**).

The review by Mitranovici and colleagues discussed the role of calcium-binding proteins S100A8/S100A9 in the physiopathology of PE, as well as the potential of these proteins as prognostic inflammatory markers for placental disfunctions in combination with established biomarkers (sFlt-1/PlGF) and as a therapeutic target in PE-related thrombosis (**Contribution 12**).

## 3. Fetal Growth Restriction

Reshetnikov and colleagues revealed 15 FGR-associated mAAM (maternal age at menarche)-related gene polymorphisms and 350 linked SNPs related to 39 genes involved in the regulation of hormone levels, ovulation cycle process, male gonad development, and vitamin D metabolism (**Contribution 13**).

The proteomic study by Starodubtseva and collaborators identified, in maternal blood plasma, 13 potential FGR-associated markers involved in plasma lipoprotein turnover, lipid metabolism, hemostasis, and immune system regulation, as well as 18 proteins specific to early or late IUGR (**Contribution 14**).

## 4. Conclusions

The articles published in this Special Issue, “Recent Molecular Research on Preeclampsia”, reflect a wide spectrum of topics in current research which are important in identifying the etiology of PE and FGR and contribute to the fundamental understanding of the nature of these syndromes. Through original research and comprehensive reviews, these works highlight the diverse mechanisms underlying the development of PE and FGR, ranging from imbalances in lipid biosynthesis and inflammation reactions to genetic mutations in a variety of genes. The presented findings can help identify the potential early biomarkers and possible therapeutic targets to develop future preventive or treatment strategies.
